# Analysis of measurable residual disease by IG/TR gene rearrangements: quality assurance and updated EuroMRD guidelines

**DOI:** 10.1038/s41375-024-02272-0

**Published:** 2024-05-14

**Authors:** Vincent H. J. van der Velden, Isabel Dombrink, Julia Alten, Giovanni Cazzaniga, Emmanuelle Clappier, Daniela Drandi, Cornelia Eckert, Eva Fronkova, Jeremy Hancock, Michaela Kotrova, Rebekka Kraemer, Mirkka Montonen, Heike Pfeifer, Christiane Pott, Thorsten Raff, Heiko Trautmann, Hélène Cavé, Beat W. Schäfer, Jacques J. M. van Dongen, Jan Trka, Monika Brüggemann, EuroMRD Consortium, Vincent H. J. van der Velden, Thorsten Raff, Jacques J. M. van Dongen

**Affiliations:** 1 Laboratory Medical Immunology, Department of Immunology, Erasmus MC, University Medical Center Rotterdam, Rotterdam, The Netherlands; 2 Department of Internal Medicine II, University Hospital Schleswig-Holstein, Kiel, Germany; 3 Department of Pediatrics, University Hospital of Schleswig-Holstein, Campus Kiel, Kiel, Germany; 4 Centro Tettamanti, Fondazione IRCCS San Gerardo dei Tintori, Monza, Italy; 5 School of Medicine, University of Milano-Bicocca, Monza, Italy; 6 Hematology Laboratory, Saint-Louis Hospital, Paris Cité University, Paris, France; 7 Université Paris-Cité, Paris, France; 8 Department of Molecular Biotechnology and health sciences, Hematology Division, University of Torino, Torino, Italy; 9 Department of Pediatric Oncology and Hematology, Charité – Universitätsmedizin Berlin, Berlin, Germany; 10 German Cancer Consortium (DKTK) and German Cancer Research Center (DKFZ), Heidelberg, Germany; 11 CLIP, Department of Pediatric Hematology and Oncology, Second Faculty of Medicine and University Hospital Motol, Charles University, Prague, Czech Republic; 12 Bristol MRD Group, Bristol Genetics Laboratory, Southmead Hospital, Bristol, UK; 13 Tyks Laboratories, Genomics Department, Turku University Hospital, Turku, Finland; 14 Department of Hematology, University Hospital Frankfurt, Frankfurt, Germany; 15 Department of Genetics, University Hospital Robert Debré, Paris, France; 16 University Children’s Hospital, Zurich, Switzerland; 17 Centro de Investigación del Cáncer-Instituto de Biología Molecular y Celular del Cáncer (CIC-IBMCC, USAL-CSIC-FICUS) and Department of Medicine, University of Salamanca, Salamanca, Spain; 18 European Scientific foundation for Laboratory Hemato Oncology (ESLHO), Zutphen, The Netherlands; 19 Department of Immunology, LUMC, Leiden, The Netherlands; 20 Present Address: Military Medical City Hospital, Doha, Qatar

**Keywords:** Genetic testing, Diagnosis

## Abstract

Minimal/measurable residual disease (MRD) diagnostics using real-time quantitative PCR analysis of rearranged immunoglobulin and T-cell receptor gene rearrangements are nowadays implemented in most treatment protocols for patients with acute lymphoblastic leukemia (ALL). Within the EuroMRD Consortium, we aim to provide comparable, high-quality MRD diagnostics, allowing appropriate risk-group classification for patients and inter-protocol comparisons. To this end, we set up a quality assessment scheme, that was gradually optimized and updated over the last 20 years, and that now includes participants from around 70 laboratories worldwide. We here describe the design and analysis of our quality assessment scheme. In addition, we here report revised data interpretation guidelines, based on our newly generated data and extensive discussions between experts. The main novelty is the partial re-definition of the “positive below quantitative range” category by two new categories, “MRD low positive, below quantitative range” and “MRD of uncertain significance”. The quality assessment program and revised guidelines will ensure reproducible and accurate MRD data for ALL patients. Within the Consortium, similar programs and guidelines have been introduced for other lymphoid diseases (e.g., B-cell lymphoma), for new technological platforms (e.g., digital droplet PCR or Next-Generation Sequencing), and for other patient-specific MRD PCR-based targets (e.g., fusion genes).

## Introduction

Minimal/measurable residual disease (MRD) monitoring in acute lymphoblastic leukemia (ALL) has been proven to be a fundamental aspect in the assessment of early response to therapy and in decision-making during and after treatment [1]. Clonal immunoglobulin (IG) and T-cell receptor (TR) gene rearrangements are highly specific markers for molecular assessment of MRD in lymphoid malignancies as they harbor long stretches of specific nucleotide sequences in the hypervariable region of the rearranged IG/TR genes. Allele-specific real-time quantitative (RQ)-PCR is still regarded as the gold standard to perform IG/TR-based MRD quantification even if new methods like digital droplet PCR (ddPCR) and amplicon-based next-generation sequencing (NGS) have been introduced [2]. Most published data on molecular MRD assessment in childhood and adult ALL are available based on IG/TR RQ-PCR MRD assessment [3–6], making this technology a highly validated method that is used in many ongoing clinical trials. Another unique feature of IG/TR-based RQ-PCR is that the vast majority of laboratories worldwide performing this type of analysis for MRD monitoring in lymphoid malignancies are interconnected within the international EuroMRD Consortium.

**Fig. 1:**
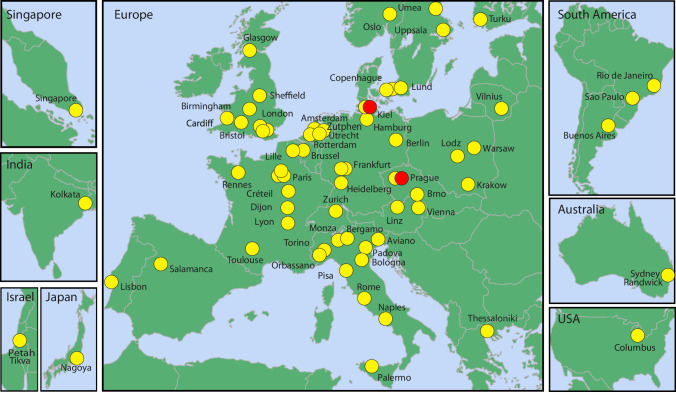
Participants of EuroMRD. The EuroMRD network, formerly known as the European Study Group on MRD detection in ALL (ESG-MRD-ALL) was established in 2001 and is currently composed of 71 MRD-PCR laboratories spread across 27 countries in Europe, Asia, Australia, North and South America. The red dots indicate the institutes of the co-chairpersons.

This consortium, formerly called the European Study Group on MRD detection in ALL (ESG-MRD-ALL), was established in 2001, aiming at the collaborative development and evaluation of IG/TR by RQ-PCR [7] and the development of guidelines for uniform interpretation of MRD data [8]. The spectrum of diseases, initially focused exclusively on ALL, has subsequently been expanded to include other lymphoid malignancies, in particular, mantle cell lymphoma (MCL) and follicular lymphoma (FL) in 2007. In addition, *BCR::ABL1* quantification in Philadelphia chromosome-positive ALL and KMT2A-rearrangements in infant ALL were added to the EuroMRD portfolio in 2008 [9]. Since 2017 EuroMRD is an independent foundation (www.EuroMRD.org) under the umbrella of the European Scientific Foundation for Laboratory Hemato-Oncology (ESLHO, www.ESLHO.org). It currently consists of 71 MRD-PCR laboratories spread across 27 countries in Europe, Asia, Australia, and North and South America (Fig. 1).

The three main goals of EuroMRD are (i) Quality: based on the organization of RQ-PCR-based MRD external quality assessment (QA) programs, (ii) Education: providing continuous education of all participating members in assay performance and data interpretation, and (iii) Innovation: the collaborative development and evaluation of new MRD strategies and techniques, such as ddPCR and NGS, and the development and update of guidelines for the interpretation of RQ-PCR-based MRD data as well as those covering new strategies and techniques.

The quality scheme currently consists of QA rounds for several disease categories (ALL and B-cell lymphoma (FL and MCL)), for different molecular markers (IG/TR gene rearrangements, *KMT2A* gene rearrangements, *BCR::ABL1, IGH::CCND1*, and *IGH::BCL2* fusion genes), and for different techniques (RQ-PCR, ddPCR, and amplicon NGS) aiming at collaborative standardization of established MRD techniques and evaluation of new MRD strategies.

Laboratories wishing to participate in the QA need to successfully apply for membership by fulfilling certain criteria e.g., extensive applicable knowledge and experience and a minimal annual intake of patients (www.EuroMRD.org). An important part of the QA is the yearly conferences, where in addition to constructive, educational discussions, regular training is offered to help reach and/or maintain a high-quality diagnostic level throughout all the participating members. In addition, the international guidelines and criteria for the interpretation of RQ-PCR-based MRD data [8] are constantly reviewed and clarified if necessary.

The aim of this paper is (1) to introduce the current EuroMRD QA scheme, focusing on the ALL (IG/TR) disease category section and (2) to present the updated guidelines for IG/TR RQ-PCR data interpretation, based on a combination of experiences and updates from the last decades of QA rounds as well as experimental data. We focused on MRD detection by IG/TR analysis in ALL patients; MRD analysis using BCR::ABL1 fusion gene transcripts is clearly different and requires specific guidelines and QA schemes [9], whereas MRD detection in lymphoma patients also has several differences compared to ALL (presence of somatic hypermutations, absence of clonal evolution, focus on fusion genes, and a low degree of tumor infiltration at diagnosis, hampering the generation of a standard curve and thereby favoring analysis by ddPCR) [10].

## Part I: QA scheme for RQ-PCR-based MRD diagnostics

### EuroMRD criteria for membership

Allele-specific RQ-PCR analysis of IG/TR gene rearrangements for quantitative MRD diagnostics in lymphoid malignancies such as ALL is a highly complex technology requiring extensive knowledge and experience. Therefore, EuroMRD has defined a “Guideline for EuroMRD participantship”, which includes the following criteria (see www.EuroMRD.org): (1) extensive knowledge of IG/TR gene rearrangements; (2) extensive experience with MRD detection via IG/TR gene rearrangements or other patient-specific PCR targets at the DNA level; and (3) position of MRD laboratory: size (personnel), relation to (inter)national treatment protocols, and minimum annual intake of new patients. After successful application and approval by the EuroMRD Board, laboratories can register via the EuroMRD web tool and participate in the QA rounds.

Information about the participating MRD-PCR laboratories is shown in Fig. 1 and is continuously updated online (www.EuroMRD.org/participants).

### QA scheme set up

**Fig. 2:**
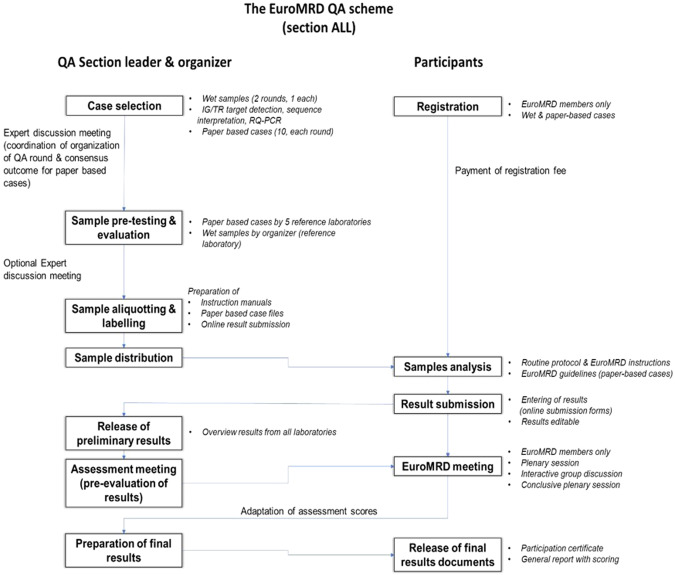
Overview of the EuroMRD quality assessment (QA). The Figure shows the various steps in the EuroMRD QA scheme for ALL for both the QA section leader & organizer (left part) as well as for the participants (right part).

All EuroMRD QA schemes are organized on a rotating basis by experienced organizer laboratories of the EuroMRD Consortium from different countries (hereafter referred to as ‘local organizer’). The scheme for the ALL (IG/TR) section consisted of two QA rounds per year, one in spring and one in autumn. The whole QA scheme process is depicted in Fig. 2.

Each round includes the so-called task 1, consisting of 10 paper-based clinical cases, and the wet lab-based task 2 or task 3, respectively. The different tasks are described in Table 1.

**Table 1:** Description of tasks in the EuroMRD QA ALL scheme.

Task no	Occurrence per year	Description of task
Task 1	2×	The correct interpretation RQ-PCR data of 10 ALL cases analyzed by IG/TR gene rearrangements. Data are presented as electronic files and participants need to analyze quantitative range, sensitivity, and MRD data.
Task 2	1×	IG/TR target detection and sequence analysis in a diagnostic sample and subsequent quantification of MRD in two to three (artificial) follow-up (FU) samples.
Task 3	1×	Analysis of provided IG/TR sequences and RQ-PCR-based MRD-quantification in two or three (artificial) FU samples.

### Sample selection

The samples originate from leftover patient materials obtained during routine care, with patients´ consent that samples can be used for scientific purposes (opt-in or opt-out depending on national regulations). The local organizers are responsible for ensuring that the way in which the samples were obtained conform to the national legal requirements for the use of patient samples, and that handling is compliant with international regulations and the Declaration of Helsinki.

For task 1, IG/TR-based RQ-PCR raw data from 10 ALL patients are selected by the local organizer, and participants should determine the quantitative range (QR), sensitivity, and MRD levels of the follow-up (FU) samples. These cases may include one to three cases that were also used in a previous QA round so that improvements over time can be analyzed. Other selected cases should mainly reflect routine cases, with the addition of one to two more challenging cases.

The samples for tasks 2 and task 3, respectively, are selected by the local organizer who is also responsible for pre-testing. For this, diagnostic DNA is extracted from bone marrow samples with known ALL infiltration and known IG/TR gene rearrangement patterns. Three dilutions of this DNA in polyclonal DNA serve as artificial MRD samples with known MRD values. All samples have a concentration of 100 ng/µl. The concentration of these samples is confirmed by both spectrophotometer measurement (e.g., NanoDrop) and control gene RQ-PCR (e.g., Albumin). Samples should be used as supplied, without adjusting of the concentration by the participants.

### Data analysis

Starting with Excel sheets in the first QA rounds, the submission and evaluation of data became more and more standardized and sophisticated over the years. Since QA round 28, an in-house developed database and a submission website (EuroMRD web tool) are provided for the submission of results and the distribution of important information. Also, the evaluation of the results and the scoring of the different tasks is done using an in-house developed database and web tool. Given the extent of the different tasks (task 1 being a paper-based task only, while task 2 includes complete marker screening and subsequent MRD analysis), the tasks are weighted differently in the scoring system.

For task 1, the reference data are determined by the consensus of five well-experienced laboratories. For task 2 and task 3, the artificial dilutions are considered as reference values. All data are presented and discussed in depth during the yearly EuroMRD meeting. After that meeting the final reference values are determined and results from participants are scored.

### Task 1: Data interpretation

For each QA round, all of the 10 paper-based cases are pre-evaluated by five experienced laboratories from the consortium (reference labs). In that way, a consensus is defined for each case.

For each case, one point is awarded for correct definition of sensitivity, QR, and classification (undef = undefined; pos = positive quantifiable; pos_blq = positive below QR; neg = negative) of all FU samples as defined according to the EuroMRD guidelines [8]. Consequently, this leads to a maximum score of 4 or 5 points per case, depending on the number of FU samples used.

### Task 2: IG/TR MRD target identification

Two points are awarded for each identified sequence (a sequence that has been detected by at least two other laboratories and conforms to the consensus interpretation) and correct nomenclature according to IMGT [11] (www.imgt.org/IMGTindex/nomenclature.php) for a maximum of three sequences. A sequence is also scored if it was not identified by any other laboratory but was used as an MRD-PCR target in an RQ-PCR assay with a QR of at least 10^–4^.

**Fig. 3:**
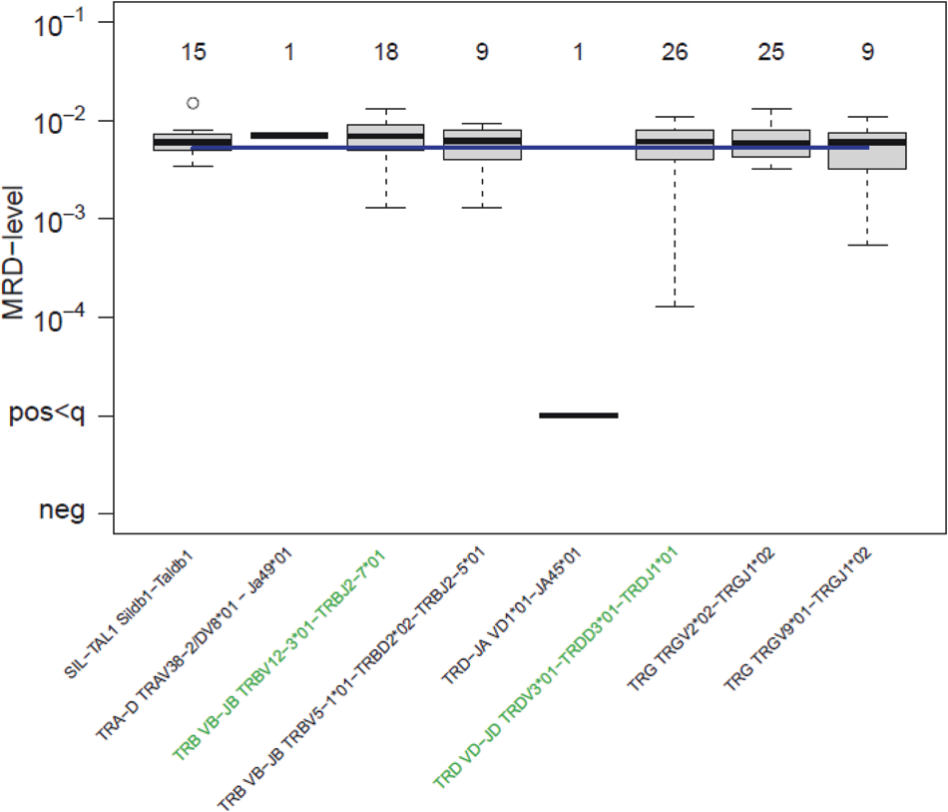
Illustration of the results obtained with Task 2 as shown in the report and certificate. The MRD levels of follow-up sample 1 were determined by the participants using different MRD-PCR targets. The names of the respective targets used for the MRD assay by the participant are colored green, the number of participants that used the respective target is written above the box plots (note: all participants will aim to analyze the FU sample using two MRD-PCR targets, therefore the sum of these numbers is about twice the number of participants). The blue line indicates the reference value.

The quantitative MRD levels are defined by the respective QA organizer by known dilutions of leukemic DNA into DNA of peripheral blood mononuclear cells of at least five healthy donors and are used as references for scoring. One point is awarded for the quantitative MRD level obtained for the two targets, as far as these levels were within a predefined upper and lower limit. The upper and lower limits were defined by a factor of 5 of the reference value for each marker and each time point (FU1-3): Limit 1 ≤ reference value ≤ Limit 2; Limit 1 = reference/sqrt(5), Limit 2 = reference*sqrt(5). The raw points of this task are multiplied with a weighting factor of 8 for the final score. Figure 3 shows an example of part of a certificate that the participant receives.

### Task 3: RQ-PCR-based MRD quantification

For task 3, the quantitative MRD levels are provided by the respective QA organizer and used as references for scoring, as already described for task 2. The raw points of this task are multiplied with a weighting factor of 10 for the final score.

### Certificates of participation and performance

Starting from the 11th QA onwards (2007), certificates of participation and performance in QA rounds are provided to the participants. Certificates of participation and performance in QA rounds are distributed after the annual meeting of the same calendar year. From QA 36 on (2019), pass and fail criteria were included in the scoring system. A certificate of performance is only provided if ≥75% of all points have been achieved (spring QA round, tasks 1 & 2; autumn QA round, tasks 1 & 3), otherwise, only a certificate of attendance is provided for the respective tasks.

**Fig. 4:**
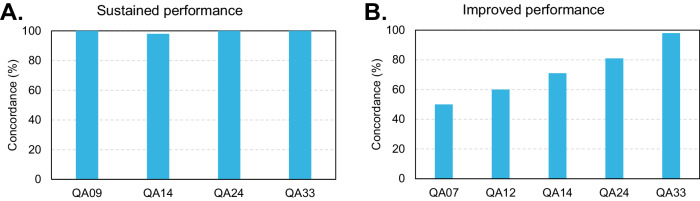
Examples of results obtained for Task 1 during various QA rounds. Sustained (**A**) and improved (**B**) performance of paper-based cases of task 1 that were analyzed during various QA rounds. Bars represent the percentages of laboratories with concordant results.

One important aspect of regular QA is the increasing knowledge and experience of the participating laboratories. To monitor the improvement of the participating laboratories, old cases from previous rounds that were used in the paper-based task 1 were sent out with new cases. As shown in Fig. 4 the performance of the participating laboratories could be drastically improved for a difficult case (Fig. 4B) and in another example, the already high performance could be sustained over time (Fig. 4A).

**Fig. 5:**
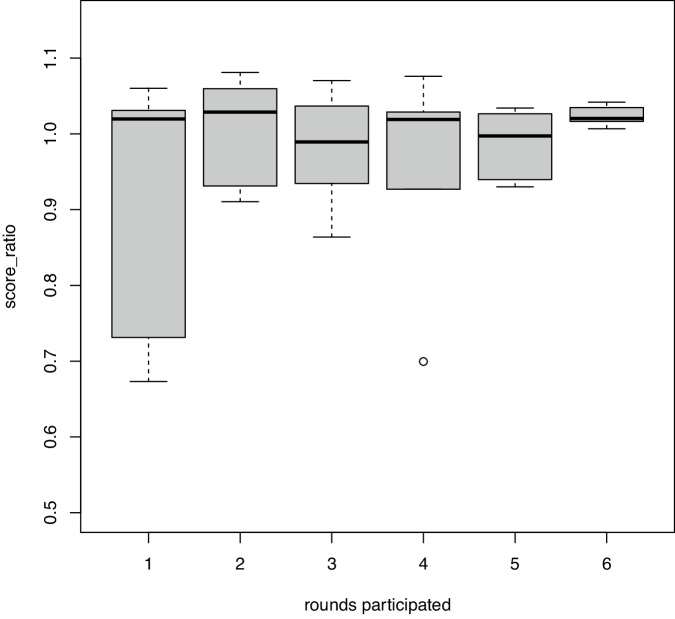
Scores obtained in various QA rounds show improved performance over time of centers that more recently joined the EuroMRD consortium. Data show the performance of six laboratories that more recently joined the ALL group. The relative performance of these labs was calculated, starting from their first participation to their sixth participation. The score ratio is defined by dividing the percentage score of the 6 newer laboratories by the mean percentage of the respective QC of all participating laboratories. Whereas the mean performance of the newer laboratories is already good from first participation onwards (showing appropriate training of these laboratories by experienced EuroMRD laboratories before entry), there are also some lower scores and some heterogeneity. There is a trend towards a more robust, more homogeneous, and good performance over time.

The annual meeting, where all the results from the last two QA rounds are discussed in depth and where educational sessions are offered, plays an important role in the improvement and the high quality of performance. All the participating laboratories, but logically particularly the newer laboratories, benefit from the co-operational, supportive structure of EuroMRD. This is illustrated by the increase in scores and reduced variability in scores of more recently entered laboratories over time, as shown in Fig. 5.

## Part II: EuroMRD guidelines and criteria for the interpretation of real-time quantitative PCR-based MRD data

### Updated guidelines

Guidelines for interpretation of RQ-PCR data include the definition of the QR and sensitivity, the definition of MRD-positivity and MRD-negativity as well as guidance on how to quantify FU samples. Fifteen years of experience and ongoing discussion at the annual EuroMRD meetings have led to the continuous revision and clarification of the guidelines for RQ-PCR data interpretation published in 2007 [8]. Therefore, the guidelines now have been formally updated and can be found in the Supplementary information, where all major changes compared to the original guideline are marked. Besides minor editing, the updates address several relevant issues.

First, the topic of appropriately selecting optimal MRD targets. According to 15 years of collective experience and increasing amounts of available NGS data, so-called public clonotypes (i.e., IG/TR gene rearrangements with specific gene usage and junctional regions which occur relatively frequently in normal lymphoid cells) should be avoided. In the same manner, targets without background amplification in the RQ-PCR should be selected whenever possible. Previous and recent studies have clearly shown that public clonotypes and/or targets with background amplification may result in false-positive MRD data and therefore should be omitted whenever possible [12–16].

A second topic is the rounding of MRD results. It was discussed and decided that for the EuroMRD QA rounds, MRD data should be reported in one significant figure and that rounding should only be done in the final step of data interpretation, till then all numbers should be used as they are. Such information and common decisions are additionally tracked and shared on the FAQ section of the EuroMRD web tool.

**Fig. 6:**
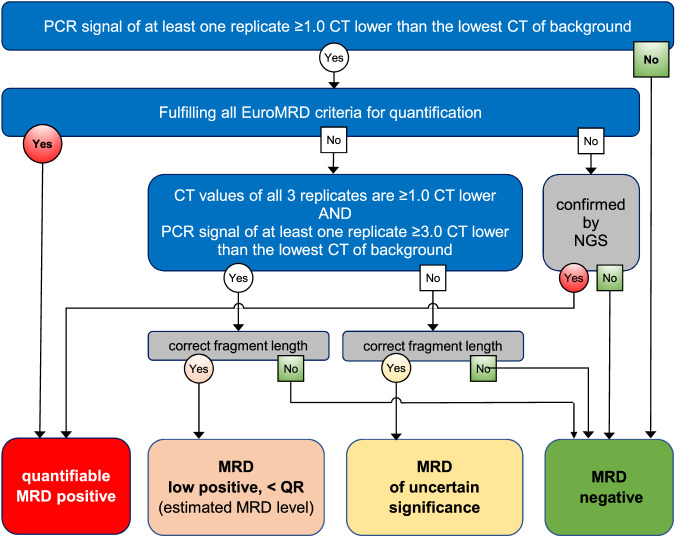
Flow chart for the interpretation of follow-up samples according to the updated EuroMRD guidelines. If fragment length analysis is not performed or inconclusive, the fragment length may be assumed to be correct.

Third, data reported as ‘positive, below QR’ were evaluated in more detail. Theoretically, Poisson distribution and non-specific amplification (background) may prevent clear distinction between truly positive and truly negative samples. Indeed, data generated after publication of the initial guidelines clearly indicate that the samples scored as ‘positive, <QR’ consists of a basket with truly MRD positive samples but also with false-positive samples, as shown by fragment length analysis and/or NGS analysis [12, 13, 17]. Based on the data reported in the accompanying manuscript by Kotrova et al. in this issue [18], the guidelines for data below the QR of the assay have been revisited (see *Supplementary information* for details). This resulted in the introduction of two new categories: ‘MRD low positive, <QR’ and ‘MRD of uncertain significance’ (Fig. 6). The *MRD low positive category* includes samples that are highly likely to be truly positive and is defined by the presence of three replicates with their *C*_T_ values ≥ 1.0 lower than the *lowest* *C*_T_ of background AND by a *C*_T_ value of at least one replicate ≥3.0 lower than the *lowest* *C*_T_ of background (Fig. 6). Samples fulfilling these criteria may, however, be re-classified based on the availability of other MRD data: (1) if NGS confirms the presence of MRD, the sample is considered MRD positive and the MRD level may be quantified using the NGS results (according to the validated assay quantification guidelines of the applied NGS assay); (2) if fragment length analysis is performed (which is strongly recommended) and does not confirm the appropriate length, the sample will be classified as MRD negative. Although by definition samples in the MRD low positive category cannot be quantified accurately, comparison with ddPCR data shows that calculated MRD levels are fair estimates of the actual MRD level as defined by ddPCR (see accompanying manuscript by Kotrova et al. in this issue [18]). Therefore, it was decided to provide this estimated MRD level in between brackets in the conclusion (e.g., MRD low positive, <10^−4^ (3 × 10^−5^)). The *“MRD of uncertain significance” category* includes all other samples with low levels of MRD positivity that cannot be quantified and that do not fulfill the criteria for MRD positivity. Also for this category, data may be re-classified to MRD negative if fragment length analysis shows incorrect amplicon length.

### Flowcharts

To facilitate the usage of the revised guidelines two flowcharts were designed. The first flowchart helps the user to define the QR and the sensitivity of every RQ-PCR assay (See Supplementary Fig. 1). The second flowchart guides the interpretation of FU samples according to the updated guidelines (See Fig. 6 or Supplementary Fig. 2).

### Reporting of MRD data

To allow standardized MRD reporting, which is a prerequisite for comparison of MRD data among clinical trials and treatment guiding in ALL, MRD result reporting needs to follow uniform guidelines. Based on routine MRD reports of several EuroMRD laboratories and legal obligations (i.e., compliance with ISO15189), recommendations for a standardized MRD report were developed. These guidelines for correct reporting of the MRD results for patient care or within clinical trials are presented in the Supplementary information.

## Discussion

Standardization and quality assessments are essential to ensure comparable MRD results between different laboratories. EuroMRD has developed guidelines for the interpretation of RQ-PCR MRD data [8]. The application of these guidelines ensures identical interpretation of MRD data between different laboratories participating in the same MRD-based clinical protocol. Furthermore, the EuroMRD guidelines facilitate the comparison of MRD data obtained in different treatment protocols, including those that evaluate new drugs, where MRD might be used as primary endpoint. Indeed, the EuroMRD guidelines are already being utilized in several related MRD intervention clinical trials which use the same induction regimen and identical MRD-based stratification, followed by treatment arms that differ per protocol. The revised guidelines presented here should further improve MRD data quality in current and future ALL treatment protocols. In addition, these guidelines have been introduced for other lymphoid diseases (e.g., B-cell lymphoma) [19], for new technological platforms (e.g., ddPCR or NGS) [19], and for other patient-specific oncogenetic MRD-PCR-based targets (e.g., fusion genes) [20–22].

Whereas most revisions in the guidelines are relatively minor, improvement in the interpretation of MRD data below the QR of the assay is certainly of major importance. Based on our experiences as well as on novel data (see accompanying manuscript by Kotrova et al. in this issue [18]), it now becomes possible to more reliably determine whether such low levels of MRD are truly positive or ambiguous. By the division of the ‘positive, below QR’ category into two new categories (‘MRD low positive, <QR’ and ‘MRD of uncertain significance’), separate criteria for protocols that aim at therapy reduction or therapy intensification become redundant and therefore the guidelines are somewhat simplified.

The here presented QA program was introduced over 20 years ago (in 2002), although with a lower number of participants and without certificates or web tool, but including two meetings a year to compare and discuss the results and to present novel developments such as analysis of new MRD-PCR targets [23–27]. This QA program has been constantly improved and updated in the last 20 years, maintaining the aim to provide comparable, high-quality MRD diagnostics. The results of our QA program confirm the competence of the participants to deliver such high-quality and reproducible MRD data.

The EuroMRD consortium has many specific features that explain its long-standing presence and success. First, the consortium includes almost all reference laboratories worldwide applying IG/TR-based RQ-PCR MRD detection in ALL. Second, the consortium serves both pediatric and adult ALL patients. Third, due to the developed guidelines and standardization, data comparison between many different trials has become possible, not only in Europe but worldwide. Fourth, EuroMRD serves as a contact for many clinical trials on ALL. Due to the longstanding and close interactions between the participants, the consortium also provides a solid base for scientific collaboration. In addition, the work done within EuroMRD can serve as an example of how to validate in-house developed tests for rare diseases according to the IVDR.

The RQ-PCR is still the gold standard for MRD detection in ALL. In addition, in a collaborative manner, new molecular MRD strategies and techniques, such as ddPCR [28] and NGS [29] have been developed and evaluated and are meanwhile integrated in the QA program. In addition to the here presented ALL (IG/TR) RQ-PCR-based MRD QA program, programs for ALL (*KMT2A*), NHL (*IGH*::*BCL2*; *IGH::CCND1*; *IGH*; *IGK*) and Ph+ALL (*BCR::ABL1*) are offered and will contribute to standardized and reproducible MRD data. In addition to molecular MRD methods, flow cytometric MRD analysis can be used in ALL. Standardized antibody panels and protocols have been designed by the EuroFlow consortium and reach sensitivities ≤10^−5^, similar to RQ-PCR [30]. Both methodologies have their advantages and disadvantages [1] and should be considered as supplementary techniques.

### Supplementary information

supplemental data

## Data Availability

The datasets generated during and/or analysed during the current study are available from the corresponding author on reasonable request.
