# Supplementary material for: Analysis of measurable residual disease by IG/TR gene rearrangements: quality assurance and updated EuroMRD guidelines
Source: Leukemia. 2024 May 14;38(6):1315–22. doi: 10.1038/s41375-024-02272-0 (PMC11147754; doi:10.1038/s41375-024-02272-0)

**Supplement**

**Analysis of measurable residual disease by IG/TR gene rearrangements: quality assurance and updated EuroMRD guidelines**

Vincent H.J. van der Velden^1*^, Isabel Dombrink^2*^, Julia Alten^3^, Giovanni Cazzaniga^4,5^, Emmanuelle Clappier^6,7^, Daniela Drandi^8^, Cornelia Eckert^9,10^, Eva Fronkova^11^, Jeremy Hancock^12^, Michaela Kotrova^2^, Rebekka Kraemer^2^, Mirkka Montonen^13^, Heike Pfeifer^14^, Christiane Pott^2^, Thorsten Raff^2a^, Heiko Trautmann^2^, Hélène Cavé^6,15^, Beat W. Schäfer^16^, Jacques J.M. van Dongen^17,18,19^, Jan Trka^11^, Monika Brüggemann^2^, EuroMRD Consortium^$^

*^1^ Laboratory Medical Immunology, Department of Immunology, Erasmus MC, University Medical Center Rotterdam, Rotterdam, The Netherlands;*

*^2^ Department of Internal Medicine II, University Hospital Schleswig-Holstein, Kiel, Germany;*

*^3^ Department of Pediatrics, University Hospital of Schleswig-Holstein, Campus Kiel, Kiel, Germany;*

*^4^ Centro Tettamanti, Fondazione TRCCS San Gerardo dei Tintori, Monza, Italy;*

*^5^ School of Medicine, University of Milano-Bicocca, Monza, Italy;*

*^6^ Hematology Laboratory, Saint-Louis Hospital, Paris Cité University, Paris, France;*

*^7^ Université Paris-Cité, Paris France*

*^8^ Department of Molecular Biotechnology and health sciences, Hematology Division, University of Torino, Italy;*

*^9^ Department of Pediatric Oncology and Hematology, Charité – Universitätsmedizin Berlin, Berlin, Germany;*

*^10^ German Cancer Consortium (DKTK) and German Cancer Research Center (DKFZ), Heidelberg, Germany;*

*^11^ CLIP, Department of Pediatric Hematology and Oncology, Second Faculty of Medicine and University Hospital Motol, Charles University, Prague, Czech Republic*

*^12^ Bristol MRD Group, Bristol Genetics Laboratory, Southmead Hospital, Bristol, United Kingdom;*

*^13^ Tyks Laboratories, Genomics Dept., Turku University Hospital, Turku, Finland;*

*^14^ Department of Hematology, University Hospital Frankfurt, Frankfurt, Germany;*

*^15^ Department of Genetics, University Hospital Robert Debré, Paris, France;*

*^16^ University Children's Hospital, Zurich, Switzerland;*

*^17^ Centro de Investigación del Cáncer-Instituto de Biología Molecular y Celular del Cáncer (CIC-IBMCC, USAL-CSIC-FICUS) and Department of Medicine, University of Salamanca, Salamanca, Spain.*

*^18^ European Scientific foundation for Laboratory Hemato Oncology (ESLHO), Zutphen, Netherlands*

*^19^ Department of Immunology, LUMC, Leiden, The Netherlands.*

^*^ These authors contributed equally

^a^ Current address: Military Medical City Hospital, Doha, Qatar

^$^ A list of all EuroMRD consortium members and their affiliations can be found at the end of the paper

**Part IIa: Analysis of measurable residual disease by IG/TR gene rearrangements: Guidelines for interpretation of real-time quantitative PCR data**

Note: The substantive changes as compared to the original publication (Van der Velden et al, Leukemia 2007) ^1^ are marked in yellow.

## Experimental set-up

A *standard curve* should be made by serially diluting the diagnostic DNA specimen in DNA obtained from mononuclear cells (MNC) from a pool of five to ten healthy donors. Due to the much easier collection of peripheral blood MNC, most laboratories within EuroMRD (previously known as European Study Group on Minimal Residual Disease in Acute Lymphoblastic Leukemia, ESG-MRD-ALL) use peripheral blood MNC. The serial dilutions should range from 10^-1^ to at least 10^-5^ (preferably with inclusion of a 5x10^-4^ step) and should be tested at least in duplicate. The first dilution step of the diagnostic sample should be based on the blast cell percentage determined in exactly the same sample (e.g. by flow cytometry), so that the 10^-1^ dilution really contains 10% blast cells. By plotting the logarithmic value of the dilution against the cycle threshold or crossing point (for simplicity, both referred to as C_T_), a standard curve can be obtained. If triplicates are used for the standard curve, all three values should be used, except for obvious technical reasons (e.g. broken capillary). Since in most MRD-based protocols a sensitivity ≤10^-4^ is required, EuroMRD recommends the use of at least 500 ng DNA per reaction.

The *threshold* of the RQ-PCR assay should always be set in the region of exponential amplification across all amplification plots. This region is depicted in the log view of amplification plots as the portion of the curve that is linear. Often the threshold automatically determined by the instrument software can be used. However, if the threshold appears to be positioned outside the linear part of (some of) the amplification curves, adjustments may be made.

To determine the *background* of the RQ-PCR assay, i.e. the non-specific amplification of comparable IG/TR gene rearrangements present in normal cells, DNA obtained from mononuclear cells from a pool of five to ten healthy donors should be used. Since non-specific amplification is generally only detected at a low level and outside the quantitative range of the RQ-PCR, non-specific amplification controls should be run at least in ≥6-fold in each RQ-PCR analysis for each IG/TR marker. The lowest C_T_ value of these non-specific amplification controls is specified as the (highest) background level. In addition, no template controls (NTC) should always be included at least in duplicate in each RQ-PCR experiment.

Analysis of *follow-up samples* should be performed in triplicate and all three values should be taken into account.

## Definition of the ‘Quantitative Range’ and the ‘Sensitivity’

The sensitivity of an RQ-PCR assay is dependent on several factors, including the type of rearrangement, the size of the junctional region, and the amount of DNA in each reaction. ^2-4^ If a relatively high proportion of leukemic cells is present, the MRD level can be reliably quantified in the majority of cases. If the level of MRD is very low the assay becomes less accurate. Thus, the variation in C_T_ values between replicates is generally less than 1.5, but this increases when the mean C_T_ value of the replicates is high (e.g. C_T_>36). ^5^ Within EuroMRD it was therefore decided to define the ‘*Quantitative Range’* and the ‘*Sensitivity*’. The ‘Quantitative Range’ reflects the part of the standard curve in which the MRD levels can be quantified reproducibly and accurately, whereas the ‘Sensitivity’ reflects the lowest MRD level that still can be detected, although not reproducibly and accurately.

To determine the ‘Quantitative Range’ and the ‘Sensitivity’ of the RQ-PCR assay, both the standard curve and the background need to be analyzed. As indicated above, non-specific amplification is often only detected at a low level and outside the quantitative range of the RQ-PCR. Consequently, an accurate determination of the non-specific amplification can be difficult. Furthermore, the non-specific amplification observed in the normal MNC DNA may not be similar to non-specific amplification present in the follow-up samples of patients as it is well known that follow-up samples can differ significantly in cellular composition, dependent on the follow-up time-point in the protocol. For example, high percentages of T cells can be found during induction therapy, ^6^ whereas high percentages of precursor-B-cells can be found post-induction and post-maintenance therapy and after hematopoietic stem cell transplantation. ^7, 8^ Consequently, due to higher frequency of IG/TR gene rearrangements, respectively, the non-specific amplification in follow-up samples may be higher than the generally used non-specific amplification control (normal PB MNC DNA). ^9^ It was decided, therefore, that within EuroMRD the ‘Quantitative Range’ should be sufficiently apart from the background, so that cellular composition-related variations in non-specific amplification would not affect MRD analysis in the ‘Quantitative Range’.

The standard curve of the RQ-PCR assay should theoretically have a slope of –3.3. However, in practice several patient-specific RQ-PCR assays may perform somewhat less efficiently, resulting in a lower slope. In addition, due to experimental variation the slope of the standard curve may sometimes be slightly higher than expected. Within EuroMRD, it was, therefore, decided to accept small deviations of the theoretical slope of –3.3.

The ‘Quantitative Range’ is defined by the lowest dilution that meets all the following criteria:

- Must give specific amplification, as determined by the shape of the amplification curve and (for the ABI Prism instruments) the multicomponent graph.
- Must give reproducible amplification: Δ C_T_ of all replicates ≤1.5;
- Must have all C_T_ values ≥3.0 lower than the *lowest* C_T_ value of the background (= amplification observed in normal MNC DNA);
- Must have a *mean* C_T_ value within a defined range from the *mean* C_T_ value of the previous 1x10^-x^ dilution point:

• 2.6 – 4.0 C_T_ between 10-fold dilutions (e.g. 10^-3^ – 10^-4^);

• 0.5 – 1.5 C_T_ between 2-fold dilutions (e.g. 10^-3^ – 5x10^-4^);

• 1.2 – 2.4 C_T_ between 3.2-fold (half log) dilutions (e.g. 10^-3^ – 3.2x10^-4^);

The ranges between two dilution points were defined based on theoretical considerations (i.e. 1 C_T_ equals a two-fold difference in amount of template) and practical applicability, as determined by analysis of over 1400 RQ-PCR assays. Note that all guidelines use one decimal place for C_T_ values; it is recommended to use the C_T_ data as they come from the RQ-PCR machine and to round up in the final step of the analysis. In the Supplement appendix 1 two examples of rounding data are presented.

The resulting standard curve, including the lowest dilution of the ‘Quantitative Range’ and *all* previous dilutions:

- Must include at least three dilution points;
- Must have a minimum range of two logs;
- Must have a slope between -3.1 and -3.9;
- Must have a correlation coefficient ≥0.98.

If an RQ-PCR does not fulfil the criteria for the ‘Quantitative Range’ and resulting standard curve, further analysis should not be performed because of the poor performance of the RQ-PCR.

In some patients it may not be possible to prepare a full standard curve (ranging from 10^-1^ to 10^-5^), for example due to a low tumor load percentage or very limited amount of diagnostic material available. In such cases, the first dilution of the standard curve can be 10^-2^ and the minimum range is allowed to be 1.5 log (10^-2^-5x10^-4^).

The ‘Sensitivity’ is the lowest dilution that meets all the following criteria:

- Must give specific amplification, as determined by the shape of the amplification curve and (for the ABI Prism instruments) the multicomponent graph;
- Must have at least one positive replicate; ΔC_T_ of the replicates is not relevant;
- Must have the *lowest* C_T_ value ≥1.0 lower than the *lowest* C_T_ value of the background (= amplification observed in normal MNC DNA);
- Must have the *lowest* C_T_ value <20 cycles from the undiluted sample or, if this undiluted sample is not included in the standard curve, from the intercept of the standard curve (representing the 10^0^ dilution).

The 20 cycles in the last criteria reflect five log steps in case of a standard curve with a maximally accepted slope (-3.9). The five log steps were chosen because the theoretical sensitivity of an RQ-PCR assay using the recommended DNA input of 500 ng/reaction (corresponding to approximately 1x10^5^ cells), is 10^-5^.

Both the ‘Quantitative Range’ and the ‘Sensitivity’ should be determined based on the results obtained in the RQ-PCR run in which the follow-up samples are analyzed.

It should be noted that the ‘Quantitative Range’ of the RQ-PCR assay should be used to determine whether the RQ-PCR assay is sufficiently sensitive (generally ≤10^-4^) for reliable quantitative MRD analysis. However, when two IG/TR targets are required per patient, some protocols allow the second target to have a lower ‘Quantitative Range’ (e.g. 5x10^-4^), if the ‘Sensitivity’ is ≤10^-4^. The required criteria for MRD-PCR targets need to be determined for each MRD-based clinical protocol since the cut-off MRD values for escalation or reduction of treatment can differ between clinical protocols.

**Figure 1** shows a flow chart how the quantitative range and sensitivity can be determined.

# **Definition of MRD-positivity and MRD-negativity in follow-up samples**

The interpretation of RQ-PCR results obtained in follow-up samples is most difficult if MRD levels are outside the ‘Quantitative Range’ of the assay. In these cases, it may not always be clear whether the signal observed is due to specific amplification from leukemic cell DNA or from non-specific amplification of normal DNA. Based on detailed analysis of RQ-PCR, fragment length and NGS data, further distinction can be made between highly likely MRD positive samples, MRD of uncertain significance samples, and MRD negative samples (see accompanying manuscript by Kotrova *et al*). By the introduction of these new categories, the previously designed separate guidelines for protocols that aim at therapy reduction and for protocols directed towards therapy intensification becomes redundant and therefore have been removed.

It is recommended to monitor two IG/TR gene rearrangements, in order to limit the chance of false-negativity due to oligoclonality or ongoing/secondary rearrangements [ref]. The highest MRD level should be used as the final MRD result and for subsequent therapeutic decisions. If more than two targets are being monitored, one should realize that this will increase the chance of having false-positive MRD data (especially if IG targets are being used, due to increased background levels in regenerating BM samples) ^9-11^.

A follow-up sample is considered to be MRD positive if:

- The C_T_ value of at least one of the three replicates is ≥1.0 C_T_ lower than the *lowest* C_T_ of background.

AND

- The C_T_ value of at least one of the three replicates is within 4.0 C_T_ from the *highest* C_T_ value of the previously defined ‘Sensitivity’;

Consequently, a follow-up sample is considered ‘MRD negative’, if no amplification is observed at all, if the lowest C_T_ value of the target is within 1 C_T_ from the lowest C_T_ of the background, or if all C_T_ values are more than four cycles separated from the highest C_T_ value of the ‘Sensitivity’.

Logically, very low MRD levels (below the ‘Quantitative Range’) should always be judged with caution; especially if only one well of the three replicates is positive. In such case, re-analysis of the doubtful sample(s) may be performed, but one should be aware that by definition the results will often not be reproducible.

### Quantitation of MRD levels in follow-up samples

Although RQ-PCR is in principle a quantitative technique, it does not mean that the data obtained can be quantified in each case. Within EuroMRD, it was decided that data can only be quantified if the MRD level is within the ‘Quantitative Range’. Outside that range, data are no longer fully reproducible and therefore cannot be quantified accurately; the only exception being samples that are confirmed MRD positive by NGS analysis. ^11-14^Thus, if RQ-PCR data are confirmed by NGS data, the quantitative results of the NGS assay (according to the validated assay quantification guidelines of the applied assay) can be reported. However, based on available data (see accompanying manuscript by Kotrova *et al*), MRD levels in samples with C_T_ values outside the quantitative range but with all replicates positive and sufficiently apart from the background, can be extrapolated from the standard curve and this information can now also reported (see below).

Furthermore, in order to obtain accurate MRD data, the RQ-PCR data obtained for the IG/TR gene rearrangements should be corrected for the amount and “amplifiability” of the DNA of the diagnosis sample and the follow-up samples. This should be done by analyzing a control gene in parallel to the IG or TR gene target. If the RQ-PCR of the control gene shows a lower amount of template than expected based on physical measurements (e.g. optical density measurement), special caution is needed since this lower value can be the result of inhibition, which can be found in a substantial number of bone marrow or peripheral blood samples (5-10%). ^15^ Addition of bovine serum albumin (BSA) prevents inhibition ^15^ and EuroMRD therefore recommends the inclusion of 0.04% BSA in all RQ-PCR reactions. Furthermore, since the addition of less amount of template will result in loss of sensitivity, the control gene values of all samples need to be within predefined ranges (e.g. 250 – 1000 ng/reaction). This is especially relevant in the analysis of follow-up samples that seem to be MRD negative. If such samples have control gene results below the predefined range, they should be reported with a clear remark about their limited sensitivity.

MRD positive follow-up samples can be quantified if:

- The *mean* C_T_ value of the replicates is ≤ than the *highest* C_T_ value of the ’Quantitative Range’;

AND

- The ΔC_T_ of the replicates is ≤1.5.

Quantitation is performed:

- Using the standard curve, excluding dilutions below the ‘Quantitative Range”, of the involved IG/TR target;
- Using the *mean* C_T_ of the triplicates of the follow-up sample;
- Correcting the MRD level according to the DNA quality/quantity of the diagnostic sample and the follow-up sample as determined by RQ-PCR of the control gene.

For MRD positive samples that *cannot* be quantified (i.e. ΔC_T_ of the replicates >1.5 and/or mean C_T_ value outside ‘Quantitative Range’):

A sample is considered to be ‘MRD low positive, < quantitative range’ if:

- The C_T_ values of all three replicates are ≥1.0 C_T_ lower than the *lowest* C_T_ of background

AND

- The C_T_ value of at least one of the three replicates is ≥3.0 C_T_ lower than the *lowest* C_T_ of background.

AND

- The C_T_ value of at least one of the three replicates is within 4.0 C_T_ from the *highest* C_T_ value of the previously defined ‘Sensitivity’;

AND

- Fragment length analysis (if performed, which is strongly recommended) confirms appropriate PCR product length (expected length or expected length + 3 nucleotides) ^11, 14, 16^.

If the mean C_T_ value of replicates is outside ‘Quantitative Range’: data should be presented as “MRD low positive, <QR” (e.g. MRD low positive, <10^-4^). If the mean C_T_ value of the replicates is within the ‘Quantitative Range’ but the ΔC_T_ of the replicates >1.5: one should aim to repeat the analysis, because the key data of the RQ-PCR assay allow a quantification on this MRD level, and only pipetting errors prevent this quantification. If such a repeat is not possible, e.g. due to a too low DNA quantity, the sample has to be formally classified as "not quantifiable for technical reasons". However, since there might be more detrimental clinical impact to report a "not quantifiable for technical reasons" than to report a quantified result with a possible slight inaccuracy, one could report such MRD result (using the mean MRD value of the replicates) with a remark about the less accurate quantification.

Since available data show that the MRD levels in the ‘positive, below quantitative range’ samples (as defined by these updated guidelines) can be extrapolated from the standard curve (see accompanying manuscript by Kotrova *et al*), the estimated MRD level is reported in between brackets, e.g. MRD low positive, <10^-4^ (6x10^-5^).

A sample is considered to be ‘MRD of uncertain significance’ if:

- The C_T_ value of at least one of the three replicates is ≥1.0 C_T_ lower than the *lowest* C_T_ of background.

AND

- The C_T_ value of at least one of the three replicates is within 4.0 C_T_ from the *highest* C_T_ value of the previously defined ‘Sensitivity’.

Also MRD positive samples that do not fulfill the criteria for quantification or for the ‘MRD low positive, < quantitative range’ category, should be considered as ‘MRD of uncertain significance’. If fragment length analysis is performed and does not confirm the appropriate size of the PCR product, the sample should be classified as MRD negative.

For samples that cannot be quantified, the quality/quantity of the DNA should always be checked by RQ-PCR analysis of the control gene. These control gene data should be within the predefined acceptable range, but are not used for correcting the MRD level.

All MRD data (either quantitative, positive below quantitative range, MRD of uncertain significance, or negative) should always be reported together with the ‘Quantitative Range’ and the ‘Sensitivity’ of the assay.

**Figure 2** shows a flow chart for the interpretation of follow-up samples.

**Part IIb: Analysis of measurable residual disease by IG/TR gene rearrangements: Guidelines for reporting MRD data**

**Essential content according to ISO 15189 norm** ^17^**:**

Lab info

- Name of the lab, including contact information (e-mail address or phone number)

Diagnostic assessment

- Clear name of the diagnostic assessment, if applicable the analytical methods (e.g. MRD analysis by qPCR)

Requesting person

- Name and contacts details of requesting person

Patient

- Identification: Name, Date of Birth or patient other identifier (trial /protocol ID/lab patient ID)

Sample(s) assessed

- Date of collection
- Type of material (bone marrow, blood, etc.)

Evaluation/interpretation

- Technical: yes
- Clinical: possible

Critical comments

- Reduced quality of the sample etc. and description of the impact on the results, if necessary

Other essential information

- Names/signatures of the person(s) (four eye principle) who checked and approved the report (not necessary that this is done by a medical doctor)
- Date of the finalization of the report
- Page numbers (page 1 of 2 etc.)
- Patient identifier on each page

Accreditation logo

**Essential MRD related information:**

Assay info

- Clonal markers used for the analysis: e.g. V(D)J names, KMT2A rearrangements, *IGH::BCL2*
- Quantitative range of each marker
- Sensitivity of each marker

Quantitative MRD results

- No digits after the comma/dot; no % 🡪 2x10^-3^ (but not 2.2x10^-3^ and not 0.22%)

Non-quantifiable MRD results

- Positive, below QR <10^-4^ positive, not quantifiable (but not 1E-06 or 10^-6^)

Negative MRD results

- Negative or <10^-5^ negative (but not 1E-08 or 10^-8^)

Maximal MRD levels of all evaluated targets

**Optional additional useful information:**

Lab Info

- Logo of the lab

Treatment protocol

- Trial or/and protocol ID and name

Disease

- ALL, lineage: BCP- or T-ALL
- Initial diagnosis or relapse diagnosis

Diagnosis sample (used for MRD marker identification and standard curve)

- Date of collection, sample ID or blast percentage

Samples assessed

- Date and of arrival
- Date of analysis
- Date of DNA extraction
- Time point during treatment

Assay info

- Explanation of terms (such as quantitative range, sensitivity, positive not quantifiable) and cite the respective paper ^1^ and its update (part IIa)
- Explanation of technology or/and sample processing, statement if analyzed according to guidelines

Cumulative MRD results

- In a table or in a figure

External services

- Involvement of external services (sequencing, e.g. *KMT2A* breakpoint sequencing by Diagnostic Center of Acute Leukemia (DCAL), Frankfurt)

Separate report for TR/IG markers identified at initial diagnosis /relapse

**Layout:**

- According to national, local requirements

**EuroMRD consortium**

Members of the EuroMRD consortium are:

Switzerland: Prof. Dr. B.W. Schäfer (Chairman) and Dr. S. Kubetzko, University Children's Hospital, Zurich, CH; The Netherlands: Dr. V.H.J. van der Velden and P. Hoogeveen, Erasmus MC, Rotterdam, NL; Prof. Dr. J.J.M. van Dongen (Founding Chairman), LUMC, Leiden, NL; Prof. Dr. C.E. van der Schoot and Dr. C. Homburg, Sanquin Research, Amsterdam, NL; Dr. E. Sonneveld, Princess Máxima Center for pediatric oncology, Utrecht, NL; United Kingdom: Dr. J. Hancock and P Archer, Southmead Hospital, Bristol, UK; Dr. J. Blackburn, Sheffield Children’s NHS Foundation Trust, Sheffield, UK; S. Chudleigh, Royal Hospital for Sick Children Yorkhill NHS Trust, Glasgow, UK; Dr. S. Bevan, Barts Health NHS Trust, The Royal London Hospital, UK; Dr. A. Fielding and Dr. K. Zuborne Alapi, University College London, London, UK; Dr. S. Adams and Dr. J. Bartram, Great Ormond Street Hospital for Children NHS Trust, London, UK; Dr. K. Wall and Dr. J. Bryon, Birmingham Women’s Hospital, Birmingham, UK; Dr. A. Gilkes, Cardiff University, Cardiff, UK; Germany: Prof. Dr. C. Schaaf and Dr. R. Koehler, University of Heidelberg, Heidelberg, DE; Prof. Dr. M. Schrappe and Dr. J. Alten, University Hospital Schleswig-Holstein – Campus Kiel, Kiel, DE; Prof. Dr. C. Baldus, Prof. Dr. M. Brüggemann, Prof. Dr. C. Pott, University Hospital Schleswig-Holstein – Campus Kiel, Kiel, DE; Prof. Dr. P. Bader and Dr. H. Kreyenberg, Universitätsklinikum Frankfurt am Main, Frankfurt am Main, DE; Dr. H. Pfeifer, Klinikum der Johann-Wolfgang-Goethe-Universität, Frankfurt am Main, DE; Dr. C. Eckert and M. Fillies, Charité CVK, Berlin, DE; Dr. U. zur Stadt, Universitätsklinikum Eppendorf, Hamburg, DE; France: Prof. Dr. E.A. Macintyre, Dr. A. Touzart and Dr. P. Villarese, Hôpital Necker-Enfants Malades, Paris, FR; Dr. J.M. Cayuela and Prof. Dr. E. Clappier, Hematology Laboratory, Universsity Hospital Saint-Louis, Paris, FR; Prof. Dr. H. Cavé and Dr. A. Caye-Eude, and Dr. C Arfeuille, Hôpital Robert Debré, Paris, FR; Dr. N. Grardel, Centre de Biologie – Pathologie, Lille, FR; Prof. Dr. M.-H. Delfau-Larue, Hôpital Henri Mondor – CHU Creteil, Creteil, FR; Prof. Dr. E. Delabesse and Dr. N. Prade, Institut Universitaire de Cancérologie de Toulouse, Toulouse, FR; Dr. C. Pastoret and Dr. M.L. Boulland, CHU Pontchaillou, Rennes, FR; Dr. M. Callanan, Hôpital Francois Mitterand – CHU Dijon, Dijon, FR; Dr. S. Hayette, CHU de Lyon, Pierre Bénite, FR; Italy: Prof. Dr. A. Biondi and Dr. G. Cazzaniga, Centro Tettamanti, Fondazione IRCCS San Gerardo dei Tintori, Monza, IT; Prof. Dr. A. Biffi, Dr. F. Lovisa and Dr. M. Campeggio, University of Padua, Padova, IT; Dr. M. Ladetto, Dr. D. Drandi, Dr. S. Ferrero, Dr. E. Genuardi, Dr. B. Alessandria, Dr. AM Civita, University of Torino, Torino, IT; Prof. Dr. A. Rambaldi, Dr. O. Spinelli and Dr. M. Tosi, Azienda Ospedaliera Papa Giovanni XXIII, Bergamo, IT; Prof. Dr. R. Foa, Dr. L. Elia, Dr. I. Della Starza, Dr. M. Cavelli, Dr. L.A. de Novi, University of Rome, Rome, IT; Dr. A. Santoro and Dr. D. Salemi, Ospedali Riuniti Vila Sofia Cervello, Palermo, IT; Prof. Dr. B. Izzo and Dr. S. Errichiello, University of Naples, Naples, IT; Dr. C. Terragna, Dr. V. Robustelli and Dr. M. Martello, University of Bologna, Bologna, IT; Dr. C. Fava and Dr. F. Daraio, University of Turin, Orbassano, IT; Dr. S. Galimberti, F.Guerrini, C. Bono University of Pisa, Pisa, IT; Dr. V. Gattei, Dr. R. Bomben Centro di Riferimento Oncologico Aviano, Aviano, IT; Austria: Dr. S. Köhrer, Children's Cancer Research Institute, Vienna, AT; Dr. O. Zach, Ordensklinikum Linz, Linz, AT; Czech Republic: Dr. J. Trka, Dr. E. Fronkova, Dr. M. Svaton and Dr. J. Zuna, Charles University, Prague, CZ; Prof. Dr. S. Pospisilova, Dr. T. Jurcek, Dr. H. Jelinkova and Dr. K. Plevova, University Hospital Brno and Masaryk University, Brno, CZ; Dr. K. Machová Poláková, Institute of Hematology and Blood Transfusion, Prague, CZ; Belgium: Dr. M. Bakkus, UZ Brussel, Brussel, BE; Spain: Dr. R. García Sanz, Dr. M.E. Sarasquete, Dr. C. Chillon, Dr. N. Puig, Hospital Clinico Universitario, Salamanca, ES; Portugal: Dr. P. Gameiro and Dr. M. Gomes da Silva, Portugese Institute of Oncology of Lisbon, Lisbon, PT; Denmark: Dr. H.O. Madsen and Dr. C. Utoft, Rigshospitalet, Copenhagen, DK; Finland: Dr. V. Kairisto, Dr. V. Juvonen, Dr. T. Lundan and Dr. M. Montonen, Turku University Hospital, Turku, FI; Norway: Dr. H. Vålerhaugen, OUS-Radiumhospitalet, Oslo, NO; Greece: Dr. K. Fotini and Dr. T. Touloumenidou, George Papanikolaou General Hospital, Thessaloniki, GR; Poland: Dr. T. Sacha, Dr. S. Czekalska and Dr. M. Zawada, Szpital Uniwersytecki w Krakowie, Krakow, PL; Dr. K. Borg and Dr. I. Solarska, Institute of Hematology and Transfusion Medicine, Warsaw, PL; Prof. Dr. W. Mlynarski and Dr. J. Taha, Central Clinical Hospital of Medical University of Lodz, Lodz, PL; Sweden: Prof. Dr. R. Amini and Dr. M. Marincevic, Uppsala University Hospital, Uppsala, SE; Dr. M. Ehinger, Skåne University Hospital, Lund, SE; Dr. M. Hultdin, University Hospital of Norrland, Umeå, SE; Australia: Dr. T. Trahair, J. Giles and Dr. M. Henderson, Children’s Cancer Institute Australia for Medical Research, Randwick, AU; Israel: Dr. S. Israeli and Dr. K. Shirchrur,

Schneider Children’s Medical Center of Israel, Petah Tikva, IL; Singapore: Prof. Dr. A.E.J. Yeoh, VIVA-NUS Central, Singapore, SG; Japan: Dr. Y. Li Jima-Yamashita, Dr. K. Horibe and Dr. M. Sananda, Nagoya Medical Centers, Nagoya, JP; USA: Dr. S.C. Reshmi and Dr. E. Stonerock, Nationwide Children's Hospital, Columbus, Ohio, USA; Brazil: Dr. I. Bendit, Hospital das Clinicas da Faculdade de Medicina da USP, Sao Paulo, BR; Dr. I. Renault Zalcberg, Instituto Nacional do Cancer/Centro de Transplante de Medula Ossea, Rio de Janeiro, BR; Argentina: Dr. C.N. Alonso, Dr. P. Rubio and Dr. M.S. Felice, Hospital de Pediatría Garrahan, Buenos Aires, AR; India: Prof. Dr. V. Saha, Dr. D. Saha and Dr. D. Ganguli, Tata Translational Cancer Research Centre, Kolkata, IN; Lituania: Dr. M. Stoskus, Vilnius University Hospital Santaros Klinikos, Vilnius, LT.

**References**

1. van der Velden VH, Panzer-Grumayer ER, Cazzaniga G, Flohr T, Sutton R, Schrauder A*, et al.* Optimization of PCR-based minimal residual disease diagnostics for childhood acute lymphoblastic leukemia in a multi-center setting. *Leukemia* 2007 Apr; **21**(4)**:** 706-713.

2. van der Velden VHJ, Willemse MJ, van der Schoot CE, Hahlen K, van Wering ER, van Dongen JJM. Immunoglobulin kappa deleting element rearrangements in precursor-B acute lymphoblastic leukemia are stable targets for detection of minimal residual disease by real-time quantitative PCR. *Leukemia* 2002 May; **16**(5)**:** 928-936.

3. Verhagen OJ, Willemse MJ, Breunis WB, Wijkhuijs AJ, Jacobs DC, Joosten SA*, et al.* Application of germline IGH probes in real-time quantitative PCR for the detection of minimal residual disease in acute lymphoblastic leukemia. *Leukemia* 2000 Aug; **14**(8)**:** 1426-1435.

4. van der Velden VHJ, Wijkhuijs JM, Jacobs DC, van Wering ER, van Dongen JJM. T cell receptor gamma gene rearrangements as targets for detection of minimal residual disease in acute lymphoblastic leukemia by real-time quantitative PCR analysis. *Leukemia* 2002 Jul; **16**(7)**:** 1372-1380.

5. van der Velden VHJ, Hochhaus A, Cazzaniga G, Szczepanski T, Gabert J, van Dongen JJM. Detection of minimal residual disease in hematologic malignancies by real-time quantitative PCR: principles, approaches, and laboratory aspects. *Leukemia* 2003 Jun; **17**(6)**:** 1013-1034.

6. van Wering ER, van der Linden-Schrever BE, van der Velden VHJ, Szczepanski T, van Dongen JJM. T-lymphocytes in bone marrow samples of children with acute lymphoblastic leukemia during and after chemotherapy might hamper PCR-based minimal residual disease studies. *Leukemia* 2001 Aug; **15**(8)**:** 1301-1303.

7. van Lochem EG, Wiegers YM, van den Beemd R, Hahlen K, van Dongen JJM, Hooijkaas H. Regeneration pattern of precursor-B-cells in bone marrow of acute lymphoblastic leukemia patients depends on the type of preceding chemotherapy. *Leukemia* 2000 Apr; **14**(4)**:** 688-695.

8. van Wering ER, van der Linden-Schrever BE, Szczepanski T, Willemse MJ, Baars EA, van Wijngaarde-Schmitz HM*, et al.* Regenerating normal B-cell precursors during and after treatment of acute lymphoblastic leukaemia: implications for monitoring of minimal residual disease. *Br J Haematol* 2000 Jul; **110**(1)**:** 139-146.

9. van der Velden VH, Wijkhuijs JM, van Dongen JJ. Non-specific amplification of patient-specific Ig/TCR gene rearrangements depends on the time point during therapy: implications for minimal residual disease monitoring. *Leukemia* 2008 Mar; **22**(3)**:** 641-644.

10. Fronkova E, Muzikova K, Mejstrikova E, Kovac M, Formankova R, Sedlacek P*, et al.* B-cell reconstitution after allogeneic SCT impairs minimal residual disease monitoring in children with ALL. *Bone Marrow Transplant* 2008 Aug; **42**(3)**:** 187-196.

11. Kotrova M, van der Velden VHJ, van Dongen JJM, Formankova R, Sedlacek P, Bruggemann M*, et al.* Next-generation sequencing indicates false-positive MRD results and better predicts prognosis after SCT in patients with childhood ALL. *Bone Marrow Transplant* 2017 Jul; **52**(7)**:** 962-968.

12. Kotrova M, Koopmann J, Trautmann H, Alakel N, Beck J, Nachtkamp K*, et al.* Prognostic value of low-level MRD in adult acute lymphoblastic leukemia detected by low- and high-throughput methods. *Blood Adv* 2022 May 24; **6**(10)**:** 3006-3010.

13. Kotrova M, Muzikova K, Mejstrikova E, Novakova M, Bakardjieva-Mihaylova V, Fiser K*, et al.* The predictive strength of next-generation sequencing MRD detection for relapse compared with current methods in childhood ALL. *Blood* 2015 Aug 20; **126**(8)**:** 1045-1047.

14. Svaton M, Skotnicova A, Reznickova L, Rennerova A, Valova T, Kotrova M*, et al.* NGS better discriminates true MRD positivity for the risk stratification of childhood ALL treated on MRD-based protocol. *Blood* 2022 Oct 14.

15. Moppett J, van der Velden VH, Wijkhuijs AJ, Hancock J, van Dongen JJ, Goulden N. Inhibition affecting RQ-PCR-based assessment of minimal residual disease in acute lymphoblastic leukemia: Reversal by addition of bovine serum albumin. *Leukemia* 2003; **17:** 268-270.

16. Fronkova E, Svaton M, Trka J. Quality Control for IG /TR Marker Identification and MRD Analysis. *Methods Mol Biol* 2022; **2453:** 91-99.

17. ISO. Medical laboratories - Requirements for quality and competence (ISO 15189:2022). 2022. <https://www.iso.org/standard/76677.html>

**Legends to the figures**

**A.**


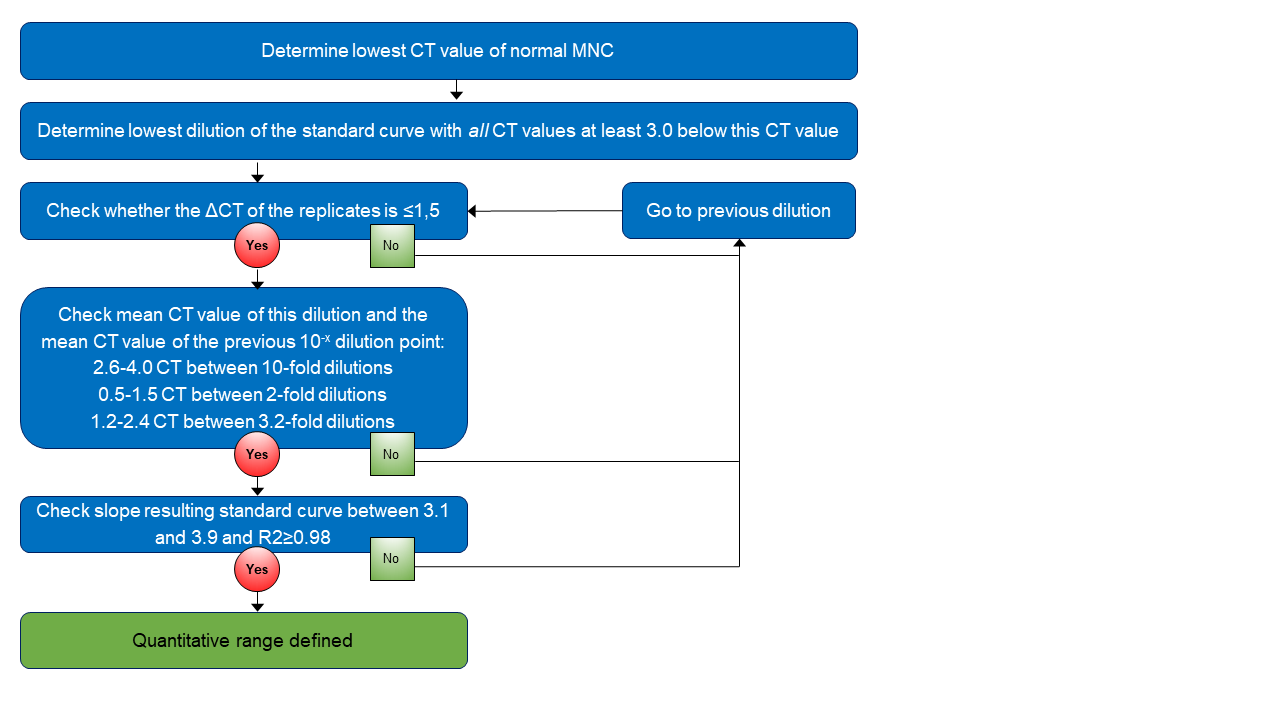


**B.**


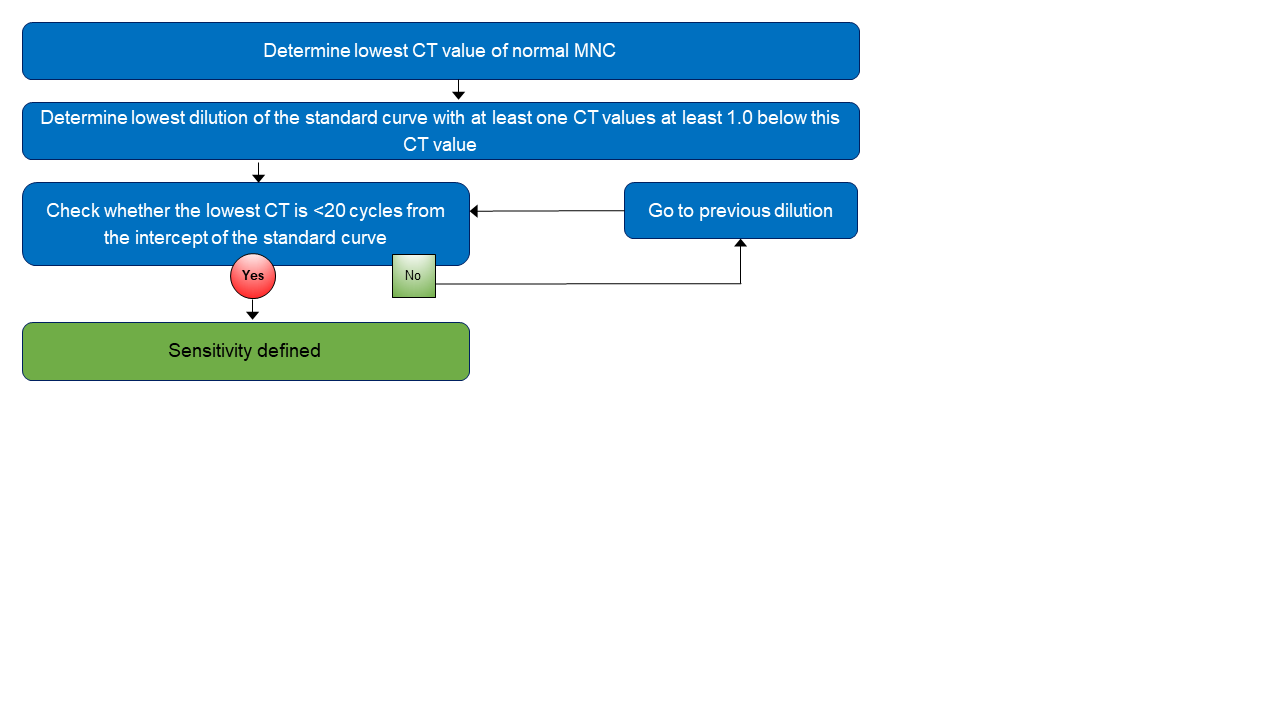


**Figure 1.** Flow chart for assessing the quantitative range (**A**) and sensitivity (**B**) of an RQ-PCR assay.


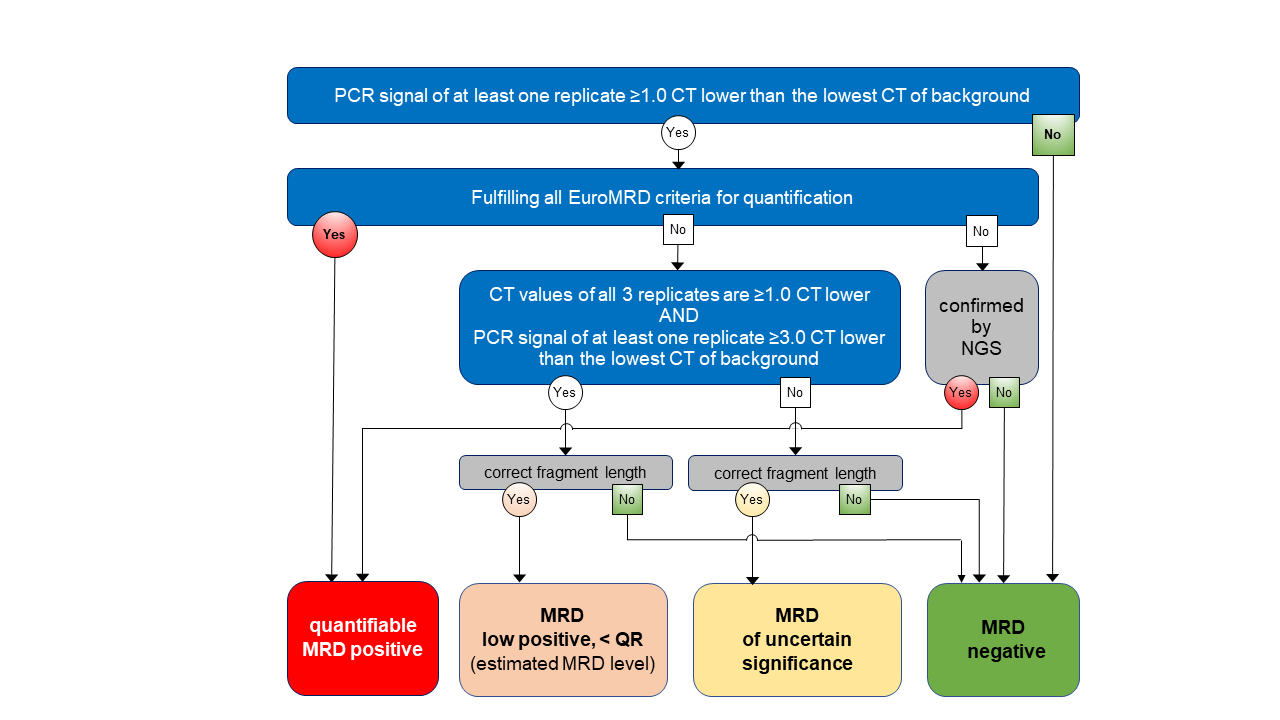


**Figure 2.** Flow chart for the interpretation of follow-up samples. If fragment length analysis is not performed or inconclusive, the fragment length may be assumed to be correct.

**Appendix 1 – Rounding issues**

**Example 1:**

The C_T_ values of the 10^-4^ dilution are reproducible (delta C_T_ <1.5) and sufficiently apart from the lowest C_T_ of the background. Calculation of the slope of a standard curve including the 10^-4^ dilution results in -3.05. Rounded to one decimal place this results in 3.0, which is outside the accepted range for the slope (-3.1 to -3.9). Therefore, the quantitative range is not 10^-4^.


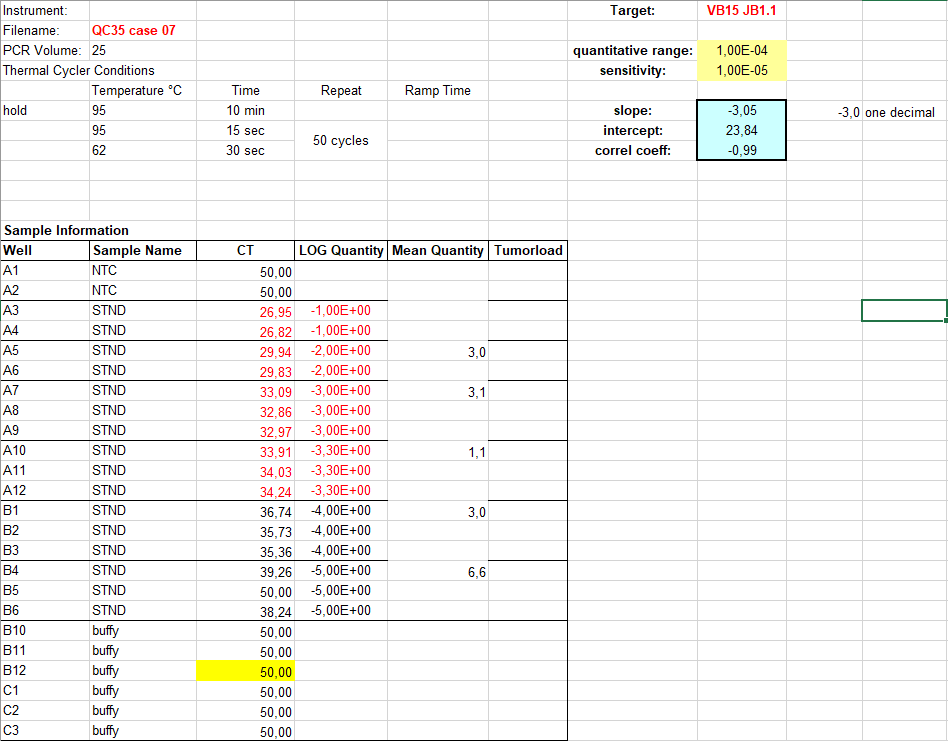


The C_T_ values of the 5x10^-4^ dilution are reproducible (delta C_T_ <1.5) and sufficiently apart from the lowest C_T_ of the background. Calculation of the slope of a standard curve including the 5x10^-4^ dilution results in -3.10. Rounded to one decimal this results in 3.1, which is inside the accepted range for the slope (-3.1 to -3.9). Therefore, the quantitative range is 5x10^-4^.


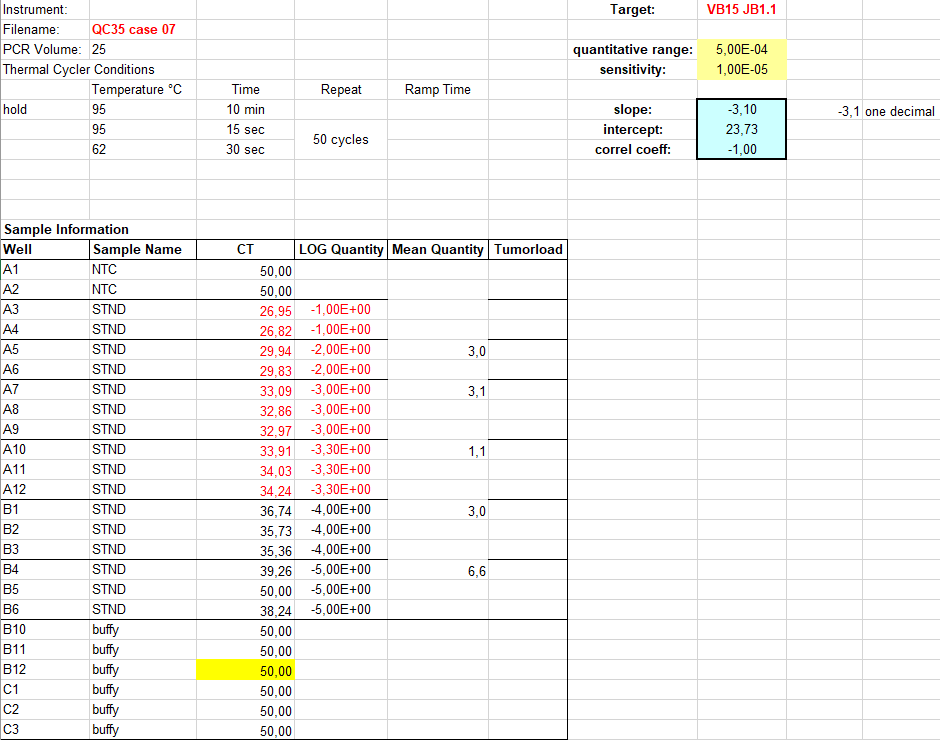


**Example 2:**

Follow up sample 2 shows non-reproducible replicates (delta C_T_ >1.5). The lowest delta C_T_ value is 41.5, which seems within 20 C_T_ from the intercept of the standard curve (21.5):


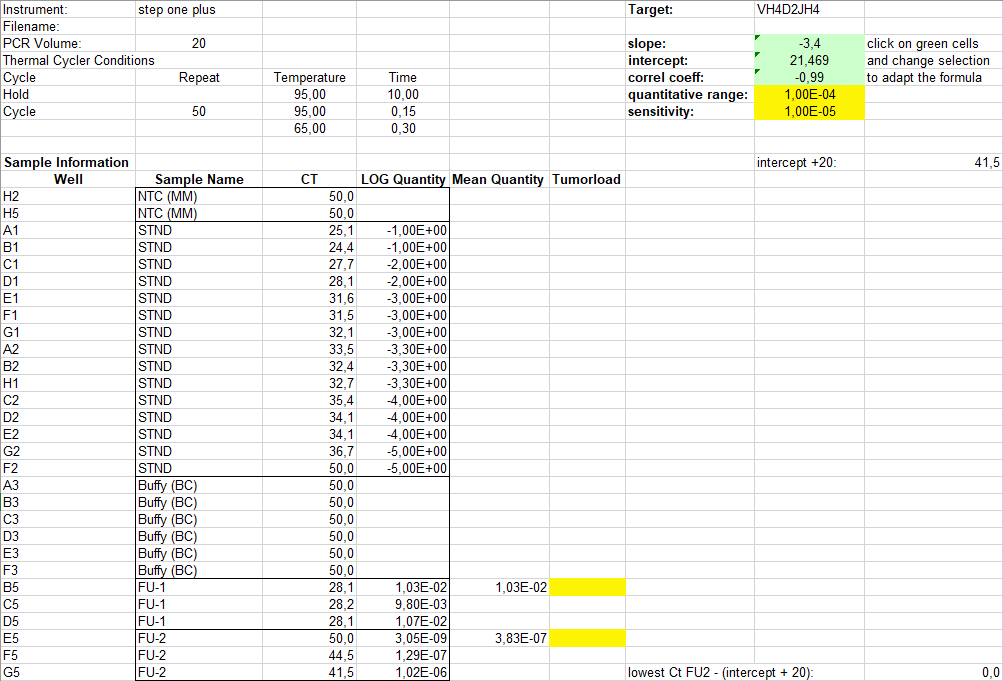


However, it the raw values from the instrument are taken into account (as is recommended), the delta C_T_ becomes >20:


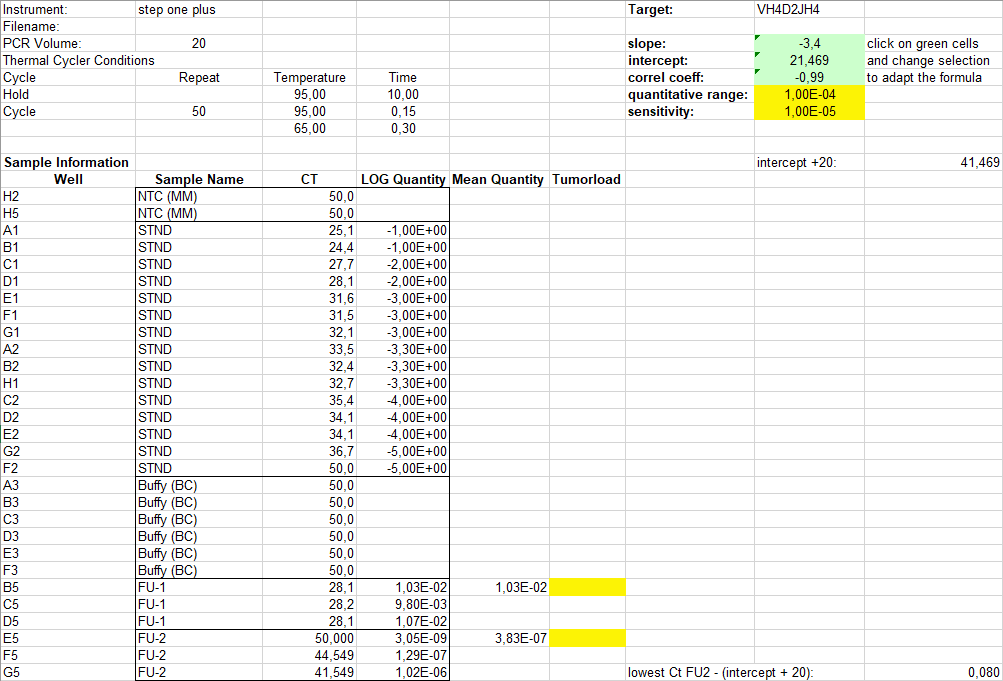

Supplement: Supplementary file 1 — supplemental data [file 41375_2024_2272_MOESM1_ESM.docx]
